# A longitudinal cohort study of acute puerperal metritis cases in Swedish dairy cows

**DOI:** 10.1186/s13028-016-0257-9

**Published:** 2016-11-10

**Authors:** Anna Ordell, Helle Ericsson Unnerstad, Ann Nyman, Hans Gustafsson, Renée Båge

**Affiliations:** 1 Department of Clinical Sciences, Swedish University of Agricultural Sciences, SE-750 07 Uppsala, Sweden; 2 Department of Animal Health and Antimicrobial Strategies, National Veterinary Institute, SE-751 89 Uppsala, Sweden; 3 Development and Services for Farmers, Växa Sverige, SE-104 25 Stockholm, Sweden

**Keywords:** Reproductive health, Postpartum, Involution, Antimicrobial treatment, Antimicrobial resistance, Fertility, Culling, Recovery, Broad spectrum

## Abstract

**Background:**

Acute puerperal metritis affects cows during the early postpartum period and causes fever, fetid vaginal discharge and general depression. The disease is severe and treatment with antimicrobials is often required. This study followed 79 Swedish dairy cows with acute puerperal metritis with registered treatment and outcome in terms of recovery. Bacteria isolated from the uterus and their susceptibility to penicillin were studied. Clinical cases were assigned by participating practitioners who examined the cows, performed uterine swab sampling, decided treatment and provided information about cow health and calving conditions. Fertility and culling data were collected from the official Swedish milk and health recording scheme. Recovery from disease was defined in four levels; as a cow that survived 1 or 4 months, was inseminated and subsequently became pregnant. Intervals from dates of first and latest calving to insemination date were studied.

**Results:**

The most common bacterial findings were a mixed culture of *Escherichia coli* and bacteria such as Gram positive cocci, *Fusobacterium necrophorum*, *Clostridium* spp. or *Trueperella pyogenes*. The Gram positive cocci, *Pasteurella* spp. and *F. necrophorum* were generally susceptible to penicillin. The majority of cows (70%) were treated with penicillin in accordance with the Swedish policy on treatment of metritis while 19% were treated with tetracycline and 8% were not treated with antimicrobials. Recovery rates were similar between treatments. Besides “calving to last insemination” interval (CLI) that was 5 days shorter than the national mean, fertility was slightly reduced compared to national means. “Calving to first insemination” interval (CFI) was 4 days longer than national mean and number of inseminations/cow increased from 1.9 to 2.1. *Escherichia coli* culture positive cows did not become pregnant to the same extent as cows without *E. coli* in the uterus (*P* = 0.046). Twin births resulted in a longer CFI (*P* = 0.034). The culling rate was generally high (42% within 300 days after occurrence of metritis), though death associated with acute disease was low (6%).

**Conclusions:**

*Escherichia coli* was the most common bacterial pathogen isolated from cases of acute puerperal metritis in the present study. This bacterium is inherently resistant to penicillin, but although most cows were treated with penicillin, death due to acute disease was low and recovery and final fertility results were acceptable. In times of emerging antimicrobial resistance and demand for prudent antimicrobial use, we suggest that penicillin is a “good enough” choice if antimicrobial treatment of acute puerperal metritis is needed.

**Electronic supplementary material:**

The online version of this article (doi:10.1186/s13028-016-0257-9) contains supplementary material, which is available to authorized users.

## Background

It is well documented that reproductive diseases have a negative effect on herd reproductive performance [1, 2]. However, the long term effect of acute puerperal metritis (APM) has not been fully investigated and recent literature is not conclusive regarding its consequences for fertility [3, 4]. After parturition, the physical barrier of the uterus is broken, the vulva is slack and the cervix dilated, which allow bacteria to invade the uterine lumen. Bacterial contamination can be detected in 50–90% of dairy cows during the first two weeks postpartum (pp) [5, 6]. In a recent Danish study, comprising 125 pp cows from five different herds, bacteria were found in all cows using a DNA-fingerprinting method [7]. In most cases, the contaminated uterus will cleanse during involution without any clinical signs [8], although occasionally, due to heavy bacterial load, failure of the immune system or other factors, bacteria remain in the uterus and cause inflammation [9]. Acute puerperal metritis affects cows up to 3 weeks pp due to profound inflammation of the uterine wall associated with fever (rectal temperature ≥39.5 °C), fetid vaginal discharge and systemic illness [10]. Common bacterial findings, in conventional culture studies, in the uterus of metritic cows are *Escherichia coli*, *Trueperella pyogenes*, and *Fusobacterium necrophorum* according to several studies [11–15].

Cows diagnosed with APM are in most cases treated with antimicrobial drugs such as penicillin, oxytetracycline, ampicillin or ceftiofur [16, 17]. The APM treatment prevalence reported in the international literature is high and varies from 15% up to 69% [18]. In Sweden, the APM treatment incidence is 0.7% according to statistics from the Swedish official milk and health recording scheme (SOMRS). However, the definition of APM varies between studies, which complicate comparisons. Systemic illness is commonly mentioned in the disease definition [3, 18, 19], but not always used as an inclusion criterion [3, 17–19].

Antimicrobials are used worldwide to treat bacterial disorders in both animals and humans but the effect has started to falter due to diminished antimicrobial susceptibility. Ceftiofur is a broad spectrum antibiotic belonging to the cephalosporins and has no withdrawal time for milk from the treated animal. It has been reported to be effective for treatment of uterine infection [19]. However, the third-generation cephalosporins are listed by the World Health Organisation (WHO) as being critically important for human medicine [20]. Each use of antimicrobial drugs contributes to the emerging resistance [21] and an association is seen between veterinary antimicrobial use and antimicrobial resistance in livestock animals [22]. The increasing resistance in zoonotic bacteria among food-producing animals has been proposed to be a potential threat to public health [21]. Thus the increased popularity of this type of drug among veterinary practitioners and farmers constitutes a risk for increasing microbial resistance.

Treatment data from Swedish practitioners’ records show that cases of APM account for 64% of the total amount of antimicrobials used in the treatment of reproductive disorders. Penicillin is used in 59% and tetracycline in 34% of all APM cases in Sweden according to the SOMRS. The Swedish Veterinary Association has stated a policy for antimicrobial use in farm animals [23]. The policy states that the use of quinolones and third and fourth-generation cephalosporins should be restricted, and that antibiotic therapy should be preceded by a diagnosis with the need for antimicrobials being evaluated in each case. According to this policy, the first-line antimicrobial therapy for most diseases in cattle is penicillin. The aim of the policy is to reduce the selective pressure on, and emergence of, resistant bacteria. It is based on literature reports of the efficacy of different treatment routines and the experience from practice that penicillin in most cases is sufficient as an antibacterial drug.

Uterine bacteria and their susceptibility to antibiotics in Swedish dairy cows with APM have not been investigated previously. The efficacy of different treatment routines in terms of how treated cows survive and remain in the herd, and whether they are inseminated and become pregnant are also unknown. The aim of this study was to investigate bacteria isolated from the uterus and their susceptibility to penicillin in cows diagnosed with APM by veterinary practitioners in Sweden, and to survey treatments used and assess the outcome in terms of survival and fertility.

## Methods

### Animal material and sampling method

Twenty practitioners from geographic areas with high number of dairy cows were instructed to sample cows with signs of APM for a longitudinal prospective cohort study. The sampling was approved by the Uppsala Animal Experiment Ethics Board (C303/11). Inclusion criteria were a fetid vaginal discharge, rectal temperature ≥39.5 °C and signs of systemic illness during the first week pp. No selection for breed, parity, housing or season was made, but cows that had already been treated with antibiotics since parturition were excluded. No treatment recommendations or instructions were given to the practitioners but they were asked to report which, if any, antibiotic substance was used, the duration of treatment, and other pharmaceutical drugs administered. The practitioners performed a clinical examination and recorded the cows’ health status at the visit, and obtained information from the farmer concerning the calving history (Table 1).

**Table 1:** Questions for the farmer about the calving history

Question	Possible answers
Calving location?	Individual calving box
	Group calving box
	On pasture
	Tied up in a pen
	In loose housing
	Other
Time for expulsion of foetal membranes?	Within 12 h pp
	12–24 h pp
	More than 24 h pp
	Still retained at visit
Calving process?	Normal without assistance
	Assistance needed
	Other

A total of 79 cows from 55 herds were sampled during a study period from November 2011 to April 2014, with most cases collected in 2012 (87%). Acute puerperal metritis cases were recorded every month of the year with most cases in November–December and February (in total 43% of the cases) and fewest cases in June–August (8%). The mean herd size was 113 cows (range 15–554) and the herds’ mean annually production was 9830 kg energy corrected milk (range 8014–11,433 kg). Among sampled cows, 28% were primiparous. The breed distribution was 53% Swedish Holstein, 43% Swedish Red and 4% crossbreed. Cows were sampled on average 4 days pp (range 1–8 days) and a retained placenta (not expelled within 24 h pp) was present in 78% of cases. According to the information given by the farmers, 67% of the cows had normal parturition (easy calving without or with slight assistance, according to codes used in reports to SOMRS), 16% twin birth, 9% abortion or premature parturition and 8% stillborn calves. Parturition took place in a group calving box (40%), in an individual calving pen (26%), tied up in a pen (23%) or in other areas (11%).

The practitioners were provided with both oral and written instructions for the sampling method, together with the sampling equipment. They were instructed to clean the external genital structures thoroughly prior to sampling with a uterine swab (Equi-Vet Uterine Culture Swab, Kruuse, Marslev, Denmark) and then take the sample according to the manufacturer’s instructions: The swab was inserted via the vulva and cervix in a sheathed tube to avoid contamination. In the uterus the sheath was withdrawn, the sample was taken from the uterine mucosa and the swab was retracted into the protective tube before withdrawal. The long swab was cut and stored in Amie’s medium (Sarstedt, Copan Italia S.p.A, Brescia, Italy) during transport by mail to the Department of Bacteriology, National Veterinary Institute, Uppsala, Sweden.

### Bacteriological culturing and antimicrobial susceptibility testing

All samples were cultured under both aerobic and anaerobic conditions and identified according to accredited conventional phenotypic methods at the Department of Bacteriology, National Veterinary Institute, Uppsala, Sweden.

The susceptibility to penicillin of isolates of Gram positive bacteria, *Pasteurella* spp. and *F. necrophorum* was tested by determination of minimum inhibitory concentration (MIC) according to the standards of the Clinical and Laboratory Standards Institute (CLSI) [24] using broth microdilution. Due to inherently low susceptibility to penicillin, Gram negative bacteria (other than *Pasteurella* spp. and *F. necrophorum*) and enterococci were not tested. For some bacterial species, changes were made compared to the CLSI standard, as described below.

Testing was performed using the panels VetMIC GP-mo (ver. 2) for Gram positive bacteria and *F. necrophorum* and VetMIC Large Animals for *Pasteurella* spp. (National Veterinary Institute, Uppsala, Sweden). The reference strain *Staphylococcus aureus* CCUG 15,915 was used as a quality control and results were within acceptable ranges. For susceptibility testing of *T. pyogenes,* colony material was suspended in 2 ml NaCl to 0.5–1 on the McFarland scale and then 10 µl were diluted in 5 ml Müller-Hinton Broth. From this dilution, 2–5 µl was inoculated on 5% horse blood agar in microdilution wells in GP-mo panels. The panels were incubated for 17–24 h at 37 °C in 5% CO_2_. For susceptibility testing of *Pasteurella* spp., 5% horse serum was added to the Müller-Hinton Broth. For susceptibility testing of *F. necrophorum*, 100 µl of a bacterial suspension with 1–5 × 10^6^ colony forming units (CFU)/ml was inoculated in each well. Susceptibility testing of other anaerobic bacteria than *F. necrophorum* was not performed.

### Survival and fertility data

Information about the sampled cows and their reproductive performance was obtained from the SOMRS, and the cows were followed from enrollment up to 18 months pp. The data obtained consisted of breed (Swedish Red/Swedish Holstein/crossbreed), parity, course of parturition (normal or assisted calving/stillbirth/twins), treatments for APM, fertility (number of inseminations, calving to first insemination interval (CFI), calving to last insemination interval (CLI), established pregnancy, fertility treatments) and culling (date, cause, euthanized/slaughter). The reasons for culling was provided by the farmer and recorded in the SOMRS.

### Statistical analyzis

Clinical records, results from bacterial culturing and antimicrobial susceptibility testing and database records on survival and fertility were statistically evaluated. Four findings indicating recovery from APM were used: (1) survival for 1 month pp (yes/no), (2) survival for four months pp (yes/no), (3) having been inseminated (yes/no) and (4) pregnancy established (yes/no). Furthermore, the two fertility variables CFI and CLI were analyzed. The studied cows’ CFI and CLI were categorized into CFI < 88 days and CFI ≥ 88 days and CLI < 130 days and CLI ≥ 130 days, respectively. The choices of 88–130 days were selected as the national means CFI and CLI in 2012 were 88–130 days, respectively [25]. Descriptive statistics were used to present the results of the distribution of the dependent (four signs of recovery and CFI ≥ 88 days and CLI ≥ 130 days) and independent variables. The independent variables were: presence of bacteria (yes/no), parity, breed, parturition (normal or assisted), twins (yes/no), retained placenta (yes/no), calving area (individual pen/group pen/tie stall/other), stillbirth (yes/no), rectal temperature >40 °C of an APM case (yes/no), treatment (only penicillin with or without non-steroidal anti-inflammatory drugs (NSAID)/only tetracycline with or without NSAID/nothing or only NSAID). Associations between the dependent variables and the independent variables were investigated using Fisher’s exact-test, univariable and multivariable logistic regression models. For the multivariable models collinearity between the independent variables was assessed pair-wise by calculation of Spearman rank correlations. If there was proof of collinearity (r ≤ 0.70) the variable with lowest *P* value in the univariable analyzis was selected. Moreover, in all the multivariable model biologically plausible two-way interactions between the main effects were tested. The model fit of the multivariable analyzes was tested by visual examination of diagnostic plots according to [26]. A *P* value of <0.05 was considered statistically significant. All statistical analyzes were performed using Stata Statistical Software (Release 11.2: College Station, TX, USA: StataCorp LP).

## Results

### Bacterial findings and antimicrobial susceptibility

Bacteria were isolated from 76 of the 79 samples (99% of the cultured samples, one had no bacterial growth and two samples were lost). Mixed growth was found in 68% of cultured samples (Table 2). Gram negative bacteria were found in 90% of the samples, and 45% contained at least one Gram positive bacterial species. Anaerobic bacteria were found in 31 samples (39%). Bacterial findings were dominated by *E. coli* often in combination with Gram positive cocci, *F. necrophorum*, and *Clostridium* spp. or *T. pyogenes*. Of the 65 *E. coli* isolates, 18% had haemolytic properties.

**Table 2:** Distribution of isolates from 76 uterine bacterial samples in pure and mixed culture

Bacteria	Isolate		Mono-culture	Mixed culture with n other bacteria			
	n	(%)		*1*	2	*3*	*4*
*Escherichia coli*	65	(86)	19	16	17	12	1
Grampositive cocci^a^	25	(33)	1	7	4	12	1
*Fusobacterium necrophorum*	18	(24)	0	0	10	8	0
*Clostridium* spp.	14	(18)	0	0	4	9	1
*Trueperella pyogenes*	16	(21)	1	6	5	3	1
Other Gram-negative^b^	9	(12)	2	3	3	1	0
*Pasturella* spp.	4	(5)	1	0	1	2	0

^a^Gram-positive cocci include various species of streptococci and enterococci

^b^Other Gram-negative bacteria include *Enterobacter* spp., *Acinetobacter* spp., *Proteus mirabilis*, *Proteus vulgaris*, *Providencia rettgeri* and *Aeromonas hydrophila*

A total of 48 isolates were tested for penicillin susceptibility. Breakpoints for resistance are not available for all bacterial species; however, MICs were low in all tested isolates (Table 3).

**Table 3:** Minimum inhibitory concentrations of penicillin for tested bacterial species

Bacterial species	MIC (mg/l)
*Trueperella pyogenes*	0. 015–0.12
*Fusobacterium necrophorum*	0.03–0.06
*Streptococcus* spp.	0.03–0.06
*Pasteurella* spp.	0.12–0.25

### Treatment

The majority of cows were treated with penicillin, often in combination with NSAID (Table 4). Tetracycline was used, by either intramuscular or intrauterine administration, in 19% of cases. Two cows were treated with both penicillin and tetracycline and one cow was treated with enrofloxacin. The mean duration of antimicrobial treatment was 5 days (range 3–6 days) regardless of substance used. No antibiotic treatment was used in six cases. NSAID was given to 68 cows (86% of all cows), while 61, six and three were given meloxicam, ketoprofen and flunixin meglumin, respectively. One cow was treated with both meloxicam and ketoprofen.

**Table 4:** Recovery related to antimicrobial and non-steroidal anti-inflammatory drugs (NSAID) treatment in number (n) and percent (%) according to four definitions of recovery

Treatment group	n	Survived 1 month		Survived 4 months		Inseminated		Pregnant	
		n	(%)	n	(%)	n	(%)	n	(%)
Penicillin	55	51	(93)	44	(80)	30	(55)	21	(38)
With NSAID	51	47	(92)	41	(80)	27	(53)	18	(35)
Tetracycline	15	12	(80)	12	(80)	9	(60)	6	(40)
With NSAID	11	9	(82)	9	(82)	7	(64)	5	(45)
No antimicrobials	6	5	(83)	5	(83)	3	(50)	2	(33)
With NSAID	4	4	(100)	4	(100)	3	(75)	2	(50)

Cows treated with both penicillin and tetracycline (n = 2) or treated with enrofloxacin (n = 1) were excluded

No significant association was found between antibiotic treatment and the four recovery parameters or the two fertility variables. There was no significant association between antibiotic treatment and parity, breed, course of calving, retained placenta or bacterial findings. The only clinical sign the veterinarians registered was rectal temperature, which was not associated with treatment. No significant association between NSAID treatment and recovery could be found.

### Fertility

Insemination was initiated for 44 cows (56%) and 31 (70%) of these subsequently became pregnant. Mean and median CFIs for these cows were 92 days (range 44–249 days) and 74.5 days (50% central range (CR): 66.5–103.5 days), respectively. In total, 26 cows had a CFI < 88 days and 18 cows had a CFI ≥ 88 days. The mean and median number of inseminations required for pregnancy was 2.1/cow (range 1–7) and 2.0 (50% CR: 1–3), respectively. Mean and median CLI was 125 days (range 65–291 days) and 112 days (50% CR: 86–146 days), respectively, for the 32 cows that became pregnant. In total, 18 cows had a CLI < 130 days and 14 cows had a CLI ≥ 130 days.

Cows with uterine *E. coli* infection had a tendency towards reduced likelihood for being inseminated compared to cows with other bacterial infections (OR = 0.24, 95% CI 0.04–1.22, *P* = 0.09) and for cows with *E. coli* infection, infection with haemolytic *E. coli* seemed to have a greater negative impact on the chance of being inseminated than for infections with non-haemolytic *E. coli* (OR = 0.32, 95% CI 0.09–1.18, *P* = 0.09). There was no significant association between the recovery parameters or fertility variables and other bacterial findings than *E. coli*, and no significant differences in treatment, culling, inseminations or pregnancy between cows with *E. coli* in either mixed culture, pure culture or negative cultures.

We found no significant association between the fertility variables and parity, breed, place of calving, retained placenta, abortion or premature parturition, stillbirth or course of calving. Cows giving birth to twins had significantly higher risk (OR = 9.6, CI 1.01–91.2, *P* = 0.05) of prolonged CFI than those with singletons. Moreover, having fever ≥40 °C on the day of examination tended to decreased the odds for subsequent pregnancy (OR = 0.32, CI 0.09–1.18, *P* = 0.09).

### Survival

In total, 43 cows (57%) were culled during the follow-up period. The mean number of days between calving and culling was 194 days (range 1–597 days). The proportion of cows culled within one month was 7/79 (9%), within four months it was 14/79 (18%) and after 18 months it was 42/79 (53%). The reported reason for culling/death was: unknown (36%), low milk production (25%), mastitis (17%), hoof/leg disorders (11%) and decreased fertility (11%).

The majority of culled cows were slaughtered, but 14 cows were euthanized or were found dead, of which half of the cases occurred within the first month pp. A significantly higher proportion of culled cows were multiparous (82%) than primiparous (18%) (*P* = 0.04 (Fisher’s exact test, could not be run using logistic regression due to too few observations in some cells). All the cows culled in the first month pp were multiparous. The risk of culling within 4 months pp tended to be higher among cows that had aborted or had a premature parturition than cows that calved at term (OR = 5.0, 95% CI 0.90–27.9, *P* = 0.07). Moreover, the risk of culling within 4 months was significantly lower among cows calving in a group calving box compared to cows calving in a single cow calving pen (OR = 0.17, 95% CI 0.03–0.93, *P* = 0.04).

A distribution table presenting associations between the recovery variables and bacterial findings, antimicrobial treatment and risk factors, bacterial findings, antimicrobial treatment and risk factors for four recovery variables and two fertility variables (survived for 1 month and 4 months, inseminated, confirmed pregnant again, CFI and CLI), including results of the statistical analyzes is included as Additional file 1: Table S1.

## Discussion

In this observational study of Swedish dairy cows with APM, *E. coli* was the most common bacterial finding and often found in combination with one or more other bacterial species (Table 2), in agreement with other reports [10, 14, 15]. These bacteria are common in the uterus after calving both in diseased and non-diseased cows. However, specific bacterial pathogens are not associated with an increased risk for developing APM [10, 11, 14]. Bacterial findings in the present study were similar to those of other studies thus implicating that bacterial species per se are an unimportant factor for the large difference observed between the prevalence of APM in Swedish statistics (<1%) and in surveys from other countries (~20%) [17, 27].

As expected, the studied cows were exposed to the known risk factors for developing APM [28], i.e. 33, 16 and 78% had a history of difficult calving, twin birth and retained placenta, respectively although the incidences were higher than in the national statistics (being 2, 3 and 1%, respectively).

The majority of cows in the study (70%) were treated with penicillin which is in accordance with the Swedish policy on the use of antibiotics recommending penicillin as first choice treatment of APM. Consequently, the use of broad-spectrum antimicrobials such as tetracycline was low (19%), and ceftiofur was not used at all. The reason behind some veterinarians’ choice not to use the recommended penicillin therapy in some cases of APM is unknown but may involve habit or clinical considerations such as herd specific historical therapy responses. The common use of broad-spectrum antimicrobials in the treatment of APM in studies from other countries may reflect treatment targeting *E. coli* as well as the availability of new broad-spectrum antibiotics (e.g. cephalosporin) without withdrawal time and reports of significant improvement in cure rates after their use [19]. However, a critical review of treating APM with ceftiofur concludes that lack of negative control groups and small samples sizes for some of the studies limits the scientific basis for this treatment [29]. One study found no significant effect of treatment with ceftiofur vs. animals treated with procaine penicillin G [16]. Another study provided some evidence that cows treated with ampicillin resulted in a more rapid clinical cure compared with cows treated with ceftiofur [30].

In the present study, all bacterial species (Gram-positive bacteria, *Pasteurella* spp. and *F. necrophorum*) that were tested for susceptibility to penicillin had low MICs, indicating clinical susceptibility (Table 3). It is known that *E. coli* is inherently resistant to penicillin, and consequently, we did not test *E. coli* isolates for susceptibility to penicillin.

The practitioners’ choice of antimicrobial treatment did not influence the recovery variables in our cohort. No significant association was found between the treatment and parity, breed, risk factors, clinical condition or bacterial findings, which may be due to the low number of observations and a low statistical power. Only 5 cows (6%) were euthanized or died while 89 and 80% survived for one and four months, respectively. This indicates a high recovery rate from APM. The main reasons for culling reported by the farmers were low milk production or mastitis for which APM may act as a pre-disposing factor [28, 31]. The total proportion of culled cows in the cohort was higher than the national mean reported in the SOMRS (57 vs. 33%).

Although a high recovery rate from APM, only 55% of the cows were inseminated (Table 4) suggesting a decline in fertility or that the farmers’ chose not to inseminate these cows again. There was a tendency that fewer *E. coli*- positive than *E. coli*-negative cows were inseminated indicating that especially this species could have a long term negative effect on fertility. However, among the 23 cows diagnosed with *E. coli* that later were inseminated and became pregnant, 17 had been treated with penicillin, which is notably considering the resistance against penicillin. Even though *E. coli* is the most commonly isolated bacterial species it may be more important to treat the other penicillin-susceptible and often tissue-invasive bacteria. New, culture-independent studies report that bacteria other than *E. coli* are the most abundant ones in the uteri of metritic cows one week pp [32], with *Fusobacteriaceae*, *Porphyromonadaceae* and *Streptococcaceae* constituting 59% of the uterine flush sample microbiota and 62% of the uterine biopsy microbiota.

An interesting finding in the present study was that of the six cows not receiving any antimicrobials, three were inseminated and two of these became pregnant (Table 4), suggesting recovery without treatment. The low number of observations and the lack of untreated controls makes it hard to draw conclusions, but our results agree well with self-cure rates in the range of 22 to 87% reported by others [17–19]. It is likely that individual cows would benefit from antimicrobial treatment; the difficulty lies in distinguishing these cows from cases where treatment is unnecessary. A recent review evaluating diagnostic methods for APM indicated that elevated rectal temperature (>39.5 °C) alone is insufficient to diagnose APM since it is also associated with factors such as parity, abnormal calving conditions and hyperketonemia [33]. It was reported that the rectal temperature exceeded 39.5 °C at least once during the first 10 days pp in 66% of healthy cows. In the present study, a tendency was seen for cows with fever ≥40.0 °C not to become pregnant as often as cows with rectal temperatures between 39.5 and 39.9 °C), regardless of antimicrobial treatment and time of the year, which indicates a more severe disease in these animals. Rectal temperature ≥40.0 °C may thus be useful as prognosis for the recovery of individual cases of APM.

In the cows subjected to insemination, the CFI was only increased by 4 days (92 vs. 88 days), and pregnancy rate/AI decreased by 7 percentage units (35 vs. 42%) compared to the national mean, while CLI was even 5 days shorter than the national mean in the cows that eventually got pregnant. This is surprisingly good despite the fact that the majority of cows were *E. coli*-positive and treated with penicillin.

## Conclusion

Although *E. coli* was the most common bacteriological finding in the uterus of cases of APM in the present study, and most cows were treated with only penicillin, acceptable recovery and fertility results were found. This supports the Swedish policy on use of antibiotics in cattle that penicillin is “good enough” as a first line therapy for treatment of bovine APM. This is also concurrent with WHO’s and OIE’s [34] demand of prudent use of antimicrobial substances in the aim to decelerate emerging antimicrobial resistance.

## Additional file

**Additional file 1: Table S1.** Table of descriptive statistics and results from the statistical analyzes.
