# Supplementary material for: A longitudinal cohort study of acute puerperal metritis cases in Swedish dairy cows
Source: Acta Vet Scand. 2016 Nov 10;58:79. doi: 10.1186/s13028-016-0257-9 (PMC5105271; doi:10.1186/s13028-016-0257-9)
Supplement: Supplementary file 1 — Additional file 1: Table S1. Table of descriptive statistics and results from the statistical analyzes. [file 13028_2016_257_MOESM1_ESM.docx]

**Additional file: Distribution of bacterial findings, antimicrobial treatment and risk factors for four recovery variables (survived for 1 and 4 months, inseminated and confirmed pregnant again), and two fertility variables (interval between calving to first insemination (CFI) and interval between calving to last insemination (CLI)) in a prospective, observational study of 79 Swedish dairy cows with puerperal metritis. The results of the statistical analyzes (Fisher´s exact-test) of associations between the recovery variables and bacterial findings, antimicrobial treatment and risk factors are also presented.**

| Recovery variable | Inseminated | | *P*-value | CFI | | *P*-value | CLI | | *P*-value | Pregnant | | *P*-value | Survived for 1 month | | *P*-value | Survived for 4 months | | *P*-value |
| --- | --- | --- | --- | --- | --- | --- | --- | --- | --- | --- | --- | --- | --- | --- | --- | --- | --- | --- |
|  | Yes | No |  | <88d | ≥88d |  | <130d | ≥130d |  | Yes | No |  | Yes | No |  | Yes | No |  |
| *Bacterial findings* | |  |  |  |  |  |  |  |  |  |  |  |  |  |  |  |  |  |
| *E. coli* |  |  |  |  |  |  |  |  |  |  |  |  |  |  |  |  |  |  |
| Yes | 34 | 31 |  | 19 | 15 |  | 12 | 11 |  | 23 | 38 |  | 58 | 7 |  | 53 | 12 |  |
| No | 9 | 2 | 0.07 | 6 | 3 | 0.71 | 5 | 3 | 0.70 | 8 | 3 | 0.05 | 11 | 0 | 0.58 | 10 | 1 | 0.68 |
| Haemolytic *E.coli* | |  |  |  |  |  |  |  |  |  |  |  |  |  |  |  |  |  |
| Yes | 4 | 8 |  | 4 | 0 |  | 2 | 1 |  | 3 | 9 |  | 11 | 1 |  | 8 | 4 |  |
| No | 39 | 25 | 0.11 | 21 | 18 | 0.13 | 15 | 13 | 1.00 | 28 | 32 | 0.21 | 58 | 6 | 1.00 | 55 | 9 | 0.20 |
| *T. pyogenes* |  |  |  |  |  |  |  |  |  |  |  |  |  |  |  |  |  |  |
| Yes | 8 | 8 |  | 3 | 5 |  | 2 | 5 |  | 7 | 7 |  | 16 | 0 |  | 14 | 2 |  |
| No | 35 | 25 | 0.58 | 22 | 13 | 0.25 | 15 | 9 | 0.20 | 24 | 34 | 0.57 | 53 | 7 | 0.33 | 49 | 11 | 0.72 |
| Gram^+^ cocci |  |  |  |  |  |  |  |  |  |  |  |  |  |  |  |  |  |  |
| Yes | 15 | 10 |  | 7 | 8 |  | 7 | 6 |  | 13 | 10 |  | 23 | 2 |  | 21 | 4 |  |
| No | 28 | 23 | 0.81 | 18 | 10 | 0.34 | 10 | 8 | 1.00 | 18 | 31 | 0.13 | 46 | 5 | 1.00 | 42 | 9 | 1.00 |
| *Fusobacterium* spp. | |  |  |  |  |  |  |  |  |  |  |  |  |  |  |  |  |  |
| Yes | 12 | 6 |  | 19 | 12 |  | 6 | 4 |  | 10 | 7 |  | 17 | 1 |  | 16 | 2 |  |
| No | 31 | 27 | 0.42 | 6 | 6 | 0.52 | 11 | 10 | 1.00 | 21 | 34 | 0.17 | 52 | 6 | 1.00 | 47 | 11 | 0.72 |
| *Klebsiella* spp. |  |  |  |  |  |  |  |  |  |  |  |  |  |  |  |  |  |  |
| Yes | 6 | 8 |  | 3 | 3 |  | 2 | 3 |  | 5 | 9 |  | 12 | 2 |  | 11 | 3 |  |
| No | 37 | 25 | 0.37 | 22 | 15 | 0.68 | 15 | 11 | 0.64 | 26 | 32 | 0.77 | 57 | 5 | 0.61 | 32 | 10 | 0.70 |
| Anaerobes |  |  |  |  |  |  |  |  |  |  |  |  |  |  |  |  |  |  |
| Yes | 16 | 15 |  | 9 | 7 |  | 7 | 6 |  | 13 | 17 |  | 28 | 3 |  | 26 | 5 |  |
| No | 27 | 18 | 0.49 | 16 | 11 | 1.00 | 10 | 8 | 1.00 | 18 | 24 | 1.00 | 41 | 4 | 1.00 | 37 | 8 | 1.00 |
| Other Gram^-^ bacteria | |  |  |  |  |  |  |  |  |  |  |  |  |  |  |  |  |  |
| Yes | 7 | 2 |  | 6 | 1 |  | 4 | 0 |  | 4 | 4 |  | 9 | 0 |  | 9 | 0 |  |
| No | 36 | 31 | 0.28 | 19 | 17 | 0.21 | 13 | 14 | 0.11 | 27 | 37 | 0.72 | 60 | 7 | 0.59 | 54 | 13 | 0.34 |
| *Pasturella* spp. |  |  |  |  |  |  |  |  |  |  |  |  |  |  |  |  |  |  |
| Yes | 2 | 2 |  | 1 | 1 |  | 2 | 0 |  | 2 | 2 |  | 3 | 1 |  | 3 | 1 |  |
| No | 41 | 31 | 1.00 | 24 | 17 | 1.00 | 15 | 14 | 0.49 | 29 | 39 | 1.00 | 66 | 6 | 0.33 | 60 | 12 | 0.54 |
|  |  |  |  |  |  |  |  |  |  |  |  |  |  |  |  |  |  |  |
| *Antimicrobial treatment* | | |  |  |  |  |  |  |  |  |  |  |  |  |  |  |  |  |
| Penicillin |  |  |  |  |  |  |  |  |  |  |  |  |  |  |  |  |  |  |
| Yes | 30 | 25 |  | 17 | 13 |  | 13 | 9 |  | 22 | 30 |  | 51 | 4 |  | 44 | 11 |  |
| No | 13 | 8 | 0.61 | 8 | 5 | 1.00 | 3 | 5 | 0.42 | 9 | 11 | 1.00 | 18 | 3 | 0.39 | 18 | 3 | 0.75 |
| Tetracycline |  |  |  |  |  |  |  |  |  |  |  |  |  |  |  |  |  |  |
| Yes | 9 | 5 |  | 5 | 4 |  | 2 | 4 |  | 6 | 7 |  | 12 | 2 |  | 12 | 2 |  |
| No | 34 | 28 | 0.57 | 20 | 14 | 1.00 | 14 | 10 | 0.38 | 25 | 34 | 1.00 | 57 | 5 | 0.61 | 50 | 12 | 1.00 |
| NSAID |  |  |  |  |  |  |  |  |  |  |  |  |  |  |  |  |  |  |
| Yes | 38 | 29 |  | 24 | 14 |  | 16 | 11 |  | 27 | 36 |  | 62 | 5 |  | 56 | 11 |  |
| No | 6 | 5 | 1.00 | 2 | 4 | 0.21 | 2 | 3 | 0.63 | 5 | 6 | 1.00 | 9 | 2 | 0.26 | 8 | 3 | 0.40 |
|  |  |  |  |  |  |  |  |  |  |  |  |  |  |  |  |  |  |  |
| *Risk factors* |  |  |  |  |  |  |  |  |  |  |  |  |  |  |  |  |  |  |
| Parity |  |  |  |  |  |  |  |  |  |  |  |  |  |  |  |  |  |  |
| First | 14 | 8 |  | 6 | 8 |  | 6 | 6 |  | 12 | 9 |  | 22 | 0 |  | 20 | 2 |  |
| Second | 9 | 9 |  | 7 | 2 |  | 3 | 4 |  | 7 | 10 |  | 14 | 4 |  | 13 | 5 |  |
| ≥ third | 21 | 17 | 0.66 | 13 | 8 | 0.27 | 9 | 4 | 0.48 | 13 | 23 | 0.33 | 35 | 3 | 0.04 | 31 | 7 | 0.32 |
| Breed |  |  |  |  |  |  |  |  |  |  |  |  |  |  |  |  |  |  |
| Swedish Holstein | 25 | 16 |  | 15 | 10 |  | 9 | 6 |  | 16 | 24 |  | 38 | 3 |  | 33 | 8 |  |
| Swedish Red | 19 | 15 |  | 11 | 8 |  | 9 | 7 |  | 15 | 16 |  | 31 | 3 |  | 29 | 5 |  |
| Cross breed | 0 | 3 | 0.13 | 0 | 0 | 1.00 | 0 | 1 | 0.71 | 1 | 2 | 0.84 | 2 | 1 | 0.34 | 2 | 1 | 0.49 |
| Normal calving | |  |  |  |  |  |  |  |  |  |  |  |  |  |  |  |  |  |
| Yes | 29 | 24 |  | 17 | 12 |  | 13 | 10 |  | 23 | 27 |  | 50 | 3 |  | 44 | 9 |  |
| No | 15 | 10 | 0.81 | 9 | 6 | 1.00 | 5 | 4 | 1.00 | 9 | 15 | 0.62 | 21 | 4 | 0.20 | 20 | 5 | 0.76 |
| Twin birth |  |  |  |  |  |  |  |  |  |  |  |  |  |  |  |  |  |  |
| Yes | 6 | 7 |  | 1 | 5 |  | 2 | 4 |  | 6 | 6 |  | 12 | 1 |  | 11 | 2 |  |
| No | 38 | 27 | 0.54 | 25 | 13 | 0.03 | 16 | 10 | 0.37 | 26 | 36 | 0.75 | 59 | 6 | 1.00 | 53 | 12 | 1.00 |
| Retained placenta |  |  |  |  |  |  |  |  |  |  |  |  |  |  |  |  |  |  |
| Yes | 33 | 28 |  | 18 | 15 |  | 14 | 12 |  | 26 | 31 |  | 56 | 5 |  | 50 | 11 |  |
| No | 11 | 6 | 0.58 | 8 | 3 | 0.48 | 4 | 2 | 0.67 | 6 | 11 | 0.58 | 15 | 2 | 0.64 | 14 | 3 | 1.00 |
| Abortion or premature parturition | | | |  |  |  |  |  |  |  |  |  |  |  |  |  |  |  |
| Yes | 2 | 4 |  | 2 | 1 |  | 1 | 1 |  | 2 | 4 |  | 6 | 0 |  | 3 | 3 |  |
| No | 42 | 30 | 0.39 | 24 | 17 | 1.00 | 17 | 13 | 1.00 | 30 | 38 | 0.69 | 65 | 7 | 1.00 | 61 | 11 | 0.07 |
| Fever |  |  |  |  |  |  |  |  |  |  |  |  |  |  |  |  |  |  |
| ≤40° | 13 | 7 |  | 7 | 6 |  | 6 | 5 |  | 11 | 7 |  | 19 | 1 |  | 18 | 2 |  |
| >40° | 11 | 11 | 0.37 | 6 | 5 | 1.00 | 3 | 4 | 1.00 | 7 | 14 | 0.11 | 19 | 3 | 0.61 | 17 | 5 | 0.41 |
| Place of calving | |  |  |  |  |  |  |  |  |  |  |  |  |  |  |  |  |  |
| Calving box | 11 | 10 |  | 7 | 8 |  | 5 | 6 |  | 11 | 10 |  | 17 | 4 |  | 16 | 5 |  |
| Group calving box | 21 | 11 |  | 15 | 6 |  | 9 | 5 |  | 14 | 16 |  | 31 | 1 |  | 30 | 2 |  |
| Tie stall | 9 | 9 |  | 3 | 6 |  | 3 | 2 |  | 5 | 12 |  | 16 | 2 |  | 14 | 4 |  |
| Other | 3 | 4 | 0.57 | 1 | 2 | 0.21 | 1 | 1 | 0.85 | 2 | 4 | 0.50 | 7 | 0 | 0.19 | 4 | 3 | 0.05 |
